# The impact of epistemic framing of teaching videos and summative assessments on students’ learning of scientific methods

**DOI:** 10.1080/09500693.2021.1998717

**Published:** 2021-12-01

**Authors:** Sibel Erduran, Olga Ioannidou, Jo-Anne Baird

**Affiliations:** Department of Education, University of Oxford, Oxford, UK

**Keywords:** Epistemic framing, scientific methods, summative assessment, teaching videos

## Abstract

The incorporation of epistemic aspects of science in science education continues to be a challenge for researchers and practitioners. The paper presents an empirical study investigating how epistemic framing of scientific methods can be incorporated in science teaching, learning and summative assessment, and what impact such framing has on student learning outcomes. The study was conducted with 969 secondary students taught by 152 teachers from a national sample in England. Teaching videos and summative assessments were framed by Brandon’s Matrix, a theoretical framework derived from the work of a philosopher of science and focusing on the diversity of scientific methods ranging from hypothesis testing to non-manipulative parameter measurement. The findings are discussed, including (a) the students’ views on the teaching videos and summative assessments, (b) the impact of the teaching videos on students’ understanding of the epistemic aspects of scientific methods and (c) students’ performance on summative assessments in the context of science topics covered in high-stakes examinations in England. The findings suggest that the students’ understanding of scientific methods significantly improved after watching the videos. Furthermore, the students’ performance on the summative assessment items indicated a high level of accuracy in responses.

## Introduction

Epistemic practices of science have been advocated as an important component of science education for numerous years (Chinn & Malhotra, 2002; Jimenez-Aleixandre et al., 2006; Kelly & Takao, 2002). Epistemic practices are the discursive practices that promote epistemic thinking and understanding (Sandoval & Reiser, 2004). For example, engagement of students in reasoning about data (Kanari & Millar, 2004), models (Justi & Gilbert, 1999) and explanations (Herman et al., 2019) is intended to promote their understanding of how scientific knowledge is constructed, refined and evaluated. Despite years of reform across the world to improve science education, the epistemic aspects of science teaching and learning continue to be of concern to many science education researchers and practitioners. Some recent curriculum standards, such as the *Next Generation Science Standards* in the USA, have integrated epistemic practices of science (NGSS Lead States, 2013). Yet the incorporation of epistemic practices in science assessments remains a challenge (National Research Council, 2014). For example, Pellegrino et al. (2014) argue that innovations in curriculum about aspects of scientific knowledge should be coupled with innovation in assessment. The authors propose a comprehensive framework for the design of assessments to illustrate how the curriculum standards promoted by NGSS can be assessed

Assessment practices, particularly summative assessments, influence how the science curriculum is experienced by students, given high-stakes examinations tend to drive teaching. Often, however, there is a disconnect between curriculum content and summative assessments, and curriculum innovation is rarely reflected in assessment design (Pellegrino, 2016).

Apart from lack of coherence between curriculum and assessment, instructional factors can play a role in how science is learned. The emergence of the Covid-19 pandemic has brought forth the necessity for online engagement in science teaching and learning, presenting new challenges for teachers and students alike. As the demand for online resources has been increasing, the question remains as to the extent to which online resources can support epistemic thinking, particularly in relation to the coordination of curriculum content and summative assessment. In this paper, we present an empirical study where teaching videos about practical work focusing on scientific methods have been framed from an epistemic perspective of scientific methods. Brandon’s Matrix was used as an epistemic framework to guide the characterisation of scientific methods (Brandon, 1994). Brandon is a philosopher of science who discussed the diversity of scientific methods ranging from hypothesis testing to non-manipulative parameter measurement. Online teaching videos and summative assessments were designed and administered to a national sample of secondary teachers and students in England. The epistemic framing of science investigations was thus achieved through the incorporation of Brandon’s Matrix into the videos about practical activities. Likewise, the summative assessments explicitly addressed the learning outcomes related to Brandon Matrix categories. Thus, by epistemic framing, we are not referring to a particular concept but rather, epistemic framing is a short-hand way of communicating how we have used an epistemic framework (i.e. Brandon’s Matrix) to explicitly re-orient and to supplement typical school practical activities and assessments. There has been widespread concern in the literature about the need to provide explicit reference to aspects of the nature of science in science teaching and learning (Khishfe, 2014; Lederman & Lederman, 2019) and our approach is consistent with such an explicit orientation, in this case about scientific methods.

The findings from the study are discussed including (a) the students’ views on the teaching videos and summative assessments, (b) the impact of the teaching videos on students’ understanding of the epistemic aspects of scientific methods and (c) students’ performance on summative assessments. The curriculum content was biology, chemistry and physics topics included in high-stakes examinations in England. Following a review of literature on epistemic practices and scientific methods as well as the interplay between curriculum and summative assessment, we describe the results and findings from an empirical study conducted with 969 students from a national sample collected in England. The reference to the interplay between curriculum and summative assessment is meant to highlight the importance of coherent alignment of content such that students’ performance on assessment tasks reflects what they are taught given a particular set of curricular objectives.

## Review of literature

### Epistemic practices and science education

Epistemic practices in science could involve a range of knowledge production and evaluation processes such as coordination of theory and evidence as well as making sense of patterns in data (Duschl & Bybee, 2014; Ford, 2015; Kelly & Licona, 2018; Manz et al., 2020; Stroupe, 2014). Furthermore, epistemic practices may refer to those cognitive and discursive activities that engage learners in the knowledge construction processes of science (Sandoval & Reiser, 2004). Science education researchers typically refer to processes of thinking and knowing that characterise particular epistemic practices such as modelling and argumentation in science (Duschl, 2008). As such, while the epistemic practices describe knowledge generation, evaluation and revision, the particular perspective through which such practices might be framed can vary. For example, some authors focus on the cultural and anthropological studies of knowledge development (Cetina-Knorr, 2007), while others may highlight cognitive psychological and philosophical accounts (Chinn et al., 2011). Conventionally in school science, students’ engagement in epistemic practices of science has been scarce (Greene & Yu, 2016). Despite years of curriculum and assessment reform across the world to improve science teaching and learning, the epistemic aspects of science teaching and learning continue to be of concern to many science education researchers (e.g. Chinn & Malhotra, 2002; Kelly & Takao, 2002; Sandoval & Reiser, 2004). A notable exception in terms of the curriculum standards is the recent depiction of scientific practices in the NGSS (NGSS Lead States, 2013), which includes learning goals related to epistemic practices of science such as argumentation and modelling.

Students’ engagement in epistemic practices can potentially contribute to the development of higher order thinking skills. The terminology of ‘higher order thinking skills’ originated in Bloom’s (1956) taxonomy and referred to seeking common standards to define educational goals and assessment. Bloom’s assumption was that student learning entails different processing levels. Bloom’s original taxonomy provided definitions for each of the six major categories in the cognitive domain (ordered from simple to complex), with the assumption that mastery of each category was a prerequisite to mastery of the next, more complex category. Since the original version of Bloom’s depiction of higher order thinking skills, numerous researchers have since proposed revised versions of his taxonomy. For example, Krathwohl (2002) proposed a revision by adding the metaknowledge category to the original categories. Earlier, Kitchener (1983) had articulated some of the details of Bloom’s categories in terms of cognitive, metacognitive and epistemic dimensions. Cognitive processing, as a first-order processing skill, includes computing, reading, perceiving and solving problems. Metacognitive processing involves monitoring the progress of the first-order tasks, and epistemic cognition is the monitoring of the cognitive and metacognitive aspects concerned with knowledge and knowing. As such, epistemic cognition entails reflection about the limits of knowing, the certainty of knowing and the criteria of knowing to account for complex monitoring.

Metacognitive knowledge was originally defined as knowledge about variables related to a person, task and/or strategy that are believed to affect cognition (Flavell, 1979). In more general terms, it is currently defined as knowledge about one’s own cognition and the control of such cognition by means of monitoring, evaluation and reflection (Barzilai & Zohar, 2012). As such, there is the component of declarative knowledge (knowing what) as well as procedural knowledge (knowing how). Such meta-knowing is composed of three main types of knowing – metacognitive, meta-strategic and epistemological knowing. Metacognitive knowing mainly refers to declarative knowledge, whereas meta-strategic knowing refers to procedural knowledge. Meta-strategic knowing refers to applying strategy knowledge about what the various thinking strategies can accomplish. Additionally, meta-strategic knowing includes task knowledge (e.g. goals and requirements of tasks), which incorporates the interrelated sub-components of knowing (Barzilai & Zohar, 2012). Finally, epistemological knowing involves knowing about knowledge in general and in relation to a person’s knowledge. In summary, the three types of knowing correspond to know-what, know-how and know-be skills (Brown & Duguid, 2001).

The types of activities in which students are engaged in school science often provide limited opportunities for the development of students’ epistemic thinking and metacognitive skills. The limitation of student engagement in epistemic practices is particularly stark in relation to the coverage of the scientific method in schools. Teaching and learning of the scientific method are typically procedural, following a linear and stepwise process. Often, it is devoid of any underpinning epistemic grounding, engaging students in mindless manipulation of apparatus and equipment, an approach referred to as the ‘cookbook problem.’ Leonard (1991) provided a description of a cookbook laboratory exercise when he wrote,

This student … is the victim of the overly prescriptive laboratory investigation, typical of those used in college introductory science courses. Such laboratory experiences tend to begin with the instructor explaining to the students, often in some detail, what will happen during the exercise in an attempt to make certain that the student will carry out the exercise ‘correctly.’ The student is then left to follow a lengthy and detailed procedure in the laboratory textbook, which will occasionally call for responses such as describing what happens with the apparatus, making a drawing, or answering a specific question in the spaces provided in the manual. The entire procedure is very prescribed, that is, the student is told what to do in a step-by-step fashion for the entire exercise. (p. 84)

Science lessons that are framed as such are fairly limited in supporting understanding of the epistemic aspects of science that the practical work is intended to promote. While there may be various aspects to the problem of epistemic framing in science lessons (e.g. students’ evaluation of scientific evidence), the focus in this paper is the context of learning of scientific methods. This is because even though the teaching and learning of scientific methods have been a goal for science education for decades, the epistemic framing of scientific investigations is still fairly rare (e.g. Manz et al., 2020). Our discussion of scientific methods will focus on a particular epistemic characterisation of scientific methods derived from an account of a philosopher of science (Brandon, 1994). The choice of this account situates our study’s reference to epistemic practices in the philosophical tradition by definition. Insofar as Brandon’s framework is used as a guiding tool for the design of instructional and assessment resources, it is a philosophical resource. However, through the transformation of the framework into teachable and concrete content , it can be said that the framework has been adapted for epistemic thinking and epistemic practice in educational contexts. The next section will articulate Brandon's work in detail following a broader context for the need to design teaching and summative assessment resources for improving students’ epistemic thinking about scientific methods.

### Scientific methods and epistemic practices

Having reviewed 70 introductory science textbooks, Blachowicz (2009) demonstrated that textbooks tend to present the scientific method as a stepwise process in a simple empiricist view of science. Woodcock (2014) discussed such ‘myths’ of the scientific method and highlighted a wide variation in the content of scientific method representations that range from 2–3 steps to 11 steps. These steps may include the following scientific processes: observing, making a hypothesis, experimenting, analysing data, confirming or rejecting the hypothesis and making conclusions. The scientific method is thus often defined as a process ‘for testing ideas and theories’ (the Merriam-Webster dictionary, n.d.).

Studies on the domain-general as well as domain-specific approaches to scientific methods illustrate the complexity and diversity of scientific methods (e.g. Frodeman, 1995). For example, Dodick et al. (2009) differentiated methodology in the historical and experimental sciences. There have been some attempts to represent a broader range of scientific methods, by frameworks alternative to ‘the scientific method’ (e.g. Lawson, 2003; Turner, 2013). Wivagg and Allchin (2002) proposed the ‘scientists’ toolbox’ to highlight the different directions that scientific investigations can take.

One philosophical framework on scientific methods that has received attention in science education is based on Brandon’s Matrix (Brandon, 1994). This framework has been adapted theoretically and applied empirically in designing empirical studies on textbooks (Wei et al., 2021 ), examination questions (Cullinane et al., 2019), undergraduate students’ understanding (Akgun & Kaya, 2020) and teachers’ (Erduran & Wooding, 2021; Erduran & Kaya, 2019; Ioannidou & Erduran, 2021) as well as students' views (El Masri et al., 2021) of scientific methods. Brandon’s Matrix (1994) presents scientific methods on the basis of whether or not they include manipulation of a variable and hypothesis testing. Based on these two parameters, scientific investigations can be categorised into four categories presented in a two-by-two table (Table 1).

**Table 1.** Representation of scientific methods (reproduced from Erduran & Dagher, 2014, p. 101).

	Experiment/observation	
	Manipulate	Not manipulate
Test hypothesis	Manipulative hypothesis test	Non-manipulative hypothesis test
	*e.g. Investigations in genetics-molecular evolution*	*e.g. Observation of Darwin’s finches*
Measure parameter	Manipulative description or measure	Non-manipulative description or measure
	*e.g. Artificial selection and breeding*	*e.g. Studies in palaeontology and developmental biology*

Brandon’s Matrix depicts the breadth of scientific methods without imposing a hierarchy between the methods. In this way, experimentation (or the manipulation of a variable) and hypothesis testing are presented as possibilities rather than necessities for a scientific investigation. An investigation can be experimental without involving hypothesis testing, while there are investigations that do not include the manipulation of a variable nor hypothesis testing. Using Brandon's framework, Erduran and Dagher (2014) give the example of artificial selection and breeding as methods that include the manipulation of variable(s) but does not involve hypothesis testing. Furthermore, some examples of palaeontology and developmental biology include investigations which do not involve the manipulation of a variable, nor the testing of a hypothesis. Brandon's Matrix can be used to illustrate the diversity in methods in specific science domains. While Brandon’s Matrix is represented in its simplistic form as a 2-by-2 table, it amounts to a more complex conceptualisation that accounts for the observation that methods in science are rarely clear cut (e.g. Allchin, 2006). While methods can be conceptualised as dichotomies, Brandon (1994) stated that it is useful to view them as components of two continua that range from testing to not testing and from manipulation to non-manipulation. A given branch of science can utilise a continuum of methods. He represented this relationship in the way depicted in Table 1, whereby investigations can be viewed as more (upper left corner) or less (lower right corner) experimental.

Brandon’s Matrix is, by definition, an epistemic framework. It was proposed by a philosopher of science (Brandon, 1994) and it utilises a language of scientific methods from a meta-perspective. While many school science activities may engage students in the activities involved in scientific methods (e.g. in hypothesis testing), explicit reflection about hypothesis testing and its relation to methods in science is not commonplace in science teaching and learning, raising concern about explicit teaching of nature of science (Abd-El-Khalick & Lederman, 2000). Indeed this concern has motivated researchers to investigate the effect of implicit versus explicit teaching of aspects of nature of science, such as scientific methods resulting in a position that explicit teaching is more effective (Khishfe, 2014). As Lederman and Lederman (2019) clarify, ‘explicit does not mean direct instruction or a lecture. The key is asking the types of questions that cause students to reflect on what they have done and concluded within a scientific investigation.’

As a theoretical framework, Brandon’s Matrix is simple enough to illustrate that the scientific method is not singular nor linear. As such, it offers flexibility in its utility as an analytical tool. For example, Cullinane et al. (2019), using Brandon’s Matrix, showed what methods underlie the practical chemistry items in high-stakes examination papers. The authors illustrated how manipulative parameter measurement dominated the examination papers and how manipulative hypothesis-testing-type questions were present in a limited capacity. This was contrary to the initial belief that manipulative hypothesis testing would be dominant, as this is often presented as ‘the scientific method’ in many science classrooms around the world. This inconsistency between the well-established idea of ‘the scientific method’ and the presentation of science methods in high-stakes examinations is a recipe for confusion, as well as leading to cookbook style procedures in the science classroom. The study, therefore, showed there is a disconnect with what is being presented as methods in science, the methods they are performing to draw conclusions from investigations and the methods of practical science that are tested on examination materials.

As a pedagogical tool, Brandon’s Matrix can be used to promote the idea that science does not follow one specific method but rather that scientific evidence is produced by the synergies between various methods and scientific fields. At the same time, the framework can be used as a metacognitive tool, as it gives the opportunity to teachers and students to reflect on the scientific investigations that are undertaken in science classrooms. For example, imagine that a student adds a universal indicator to a sample of tap water to test whether or not the sample has a pH of 7. The subsequent segment of a lesson can focus on the observations of colour and confirmation of whether or not the sample is neutral. This approach of teaching and learning, as described, is implicitly about non-manipulative parameter measurement. However, if the activity is not making explicit the method by moving beyond these observations to have discussions about what the method was, how it involved no manipulation of variables, then it would not include any epistemic framing. It would simply be about the concepts of ‘pH’ and ‘neutrality’ with implicit assumptions about acidity and alkalinity as the underpinning science concepts. Epistemic framing of the activity would be achieved when the teacher encourages the students to reflect on the nature of the methods employed in the investigation. If the teacher encouraged students through questioning to reflect on the method and how it enables understanding about neutrality, then the process would be facilitating epistemic understanding. Through repeated tasks, students would learn that the categorisation of the scientific investigations into Brandon’s Matrix is not rigid, as the matrix can be also be viewed as a continuum between ‘more experimental’ and ‘less experimental’ investigations (Erduran & Dagher, 2014). Comparison of Brandon Matrix categories would further enhance reflection.

### Interplay of curriculum and assessment

Epistemic framing of scientific methods in science education with the use of frameworks such as Brandon’s Matrix (Brandon, 1994) needs to acknowledge not only curriculum but also assessment goals. This is because the assessment has come to dominate teaching and learning in many educational settings internationally. Such dominance is particularly noteworthy when there are high stakes associated with the assessments. A high-stakes assessment is one that has direct consequences in the form of rewards or sanctions for the teachers, their institutions or the students (Madaus, 1988). Such rewards could involve students graduating to the next year with their cohort, teachers’ performance-related pay or accountability mechanisms for the school’s performance, for example. Under these circumstances, the taught curriculum is narrowed to the material that will be tested (Au, 2007; Madaus et al., 2009). Coherent alignment between curriculum and assessment clearly has benefits through clear goal-setting for instruction (Frederiksen & Collins, 1989; Shepard, 1993). Viewed positively, this has been termed measurement-driven instruction (Popham, 1987). However, it has become apparent that measurement-driven instruction has negative effects upon teaching and learning, such as the cookbook approach to teaching science lessons discussed above (e.g. Darling-Hammond, 2010; Madaus et al., 2009).

Instrumental teaching to the test without due concern for a depth of understanding has repeatedly been observed. Fragmentation of the curriculum aligned with the way in which it will be assessed has also often resulted. Such an approach leads to a superficial understanding of the curriculum, with students learning disconnected knowledge without any epistemic framework to form a deeper understanding of the connections within a discipline (Daly et al., 2012; Darling-Hammond, 2010; Darling-Hammond & Rustique-Forrester, 2005; Madaus et al., 2009; Resnick & Schantz, 2017). A proposal for countering these serious effects upon learning has been to design tests worth teaching to (Koretz, 2005). Though the notion has been much discussed, criteria for tests worth teaching to which address conventional shortcomings about coherence between curriculum and assessment have not been produced.

Our research, therefore, set out to design not only a curriculum intervention to teach students epistemic knowledge in science. Assessments were designed alongside the curriculum, in an attempt to produce *tests worth teaching to*. Design principles involved questions that would allow students to demonstrate the application of epistemic knowledge to specific science topics. Given the profound effects of assessment on learning, in turn, assessments of this design are intended to drive the desired learning about epistemic knowledge in a way that would scaffold an understanding of the variety of scientific methods in science. However, this article reports on a trial of the curriculum intervention and its associated assessments. Consequent effects of such an assessment design upon teaching could only be demonstrated if the assessments were incorporated in the assessment regime and through the study on its effects upon the taught curriculum

In the rest of the paper, we will present an empirical investigation where we traced the impact of an online teaching and summative assessment intervention on students’ understanding of Brandon’s Matrix categories. Our ultimate purpose in conducting this study was to investigate if students’ understanding of the diversity of methods used in science can be enhanced through the use of a heuristic such as Brandon’s Matrix, a simple heuristic that addresses the limitations of the standard linear scientific method represented in textbooks (Blachowicz, 2009). For the purposes of illustrating the epistemic framing of high-stakes examination questions in national tests, we chose three topics, each corresponding to the biology (osmosis), chemistry (chromatography) and physics (electromagnetic spectrum) curricula. The impact of framing of the online teaching of the topics is discussed. Furthermore, we focus on performance assessments about the physics and biology topics as they provide contrasting elements of Brandon’s Matrix: manipulative parameter measurement and non-manipulative parameter measurement. We focus on the accuracy of the key criterion of their performance: type of methods and variables. In other words, we start broadly across all subjects and illustrate the impact of curriculum design on students’ understanding, and subsequently focus on specific outcomes expressed in summative assessments that provide the contrasting aspects of Brandon’s Matrix characterisation of scientific methods. The assessment dimension of our analysis is not an exhaustive account of all Brandon’s Matrix categories but rather illustrative of an approach of establishing coherence with a given construct about types of scientific methods. In other words, our take on the assessment data is intended to highlight some example outcomes in terms of students’ performance on summative assessments across contrasting epistemic aspects of scientific methods when assessments are designed in a coherent fashion with curricular objectives.

## Methodology

The particular objective of this paper is to present an empirical study that traced the impact on students of epistemic framing of teaching videos (also referred to as online lesson videos) and summative assessments. The epistemic framing was done through the incorporation of Brandon’s Matrix as a framework that guided the production of the videos lessons and the summative assessments. The broader context of the study is a 3-year project that aimed to incorporate epistemic perspectives on practical science in science education in England. Project Calibrate was guided by a systematic approach to considerations of teaching and assessment in an effort to make them consistent in terms of content. In other words, considering there is often a disconnect in innovation in pedagogical and summative assessment practices (Pellegrino et al., 2014), the project aimed to bring coherence to what is taught and what is assessed by using a robust theoretical model on scientific methods. However, the focus was on students’ outcomes in terms of their understanding and performance of scientific methods as expressed in the pre- and post-surveys investigating student understanding of scientific methods as well as their performance on the summative assessments. In this respect, the main element of intervention is the video lessons, not the summative assessments. The summative assessments are used as an indicator of student performance to complement the survey data. In the rest of this section, then, we present the key research question driving the study, the data sources, a description of the teaching intervention (in the form of online videos) and the research instrument, which was a pre-post-survey on students’ understanding of scientific methods. The research from the larger project that has also investigated teachers’ as well as students' views of scientific methods reported elsewhere (e.g. El Masri et al., 2021). The project resources including the teaching videos and the summative assessments can be downloaded at https://projectcalibrate.web.ox.ac.uk.

### Research questions

The study was guided by the following research questions:

1. What is the impact of epistemic framing of teaching videos about scientific methods on students’ understanding of scientific methods?
2. How do students perform on summative assessments about scientific methods, having watched epistemically framed teaching videos about scientific methods?

### Data sources

**Figure 1.**
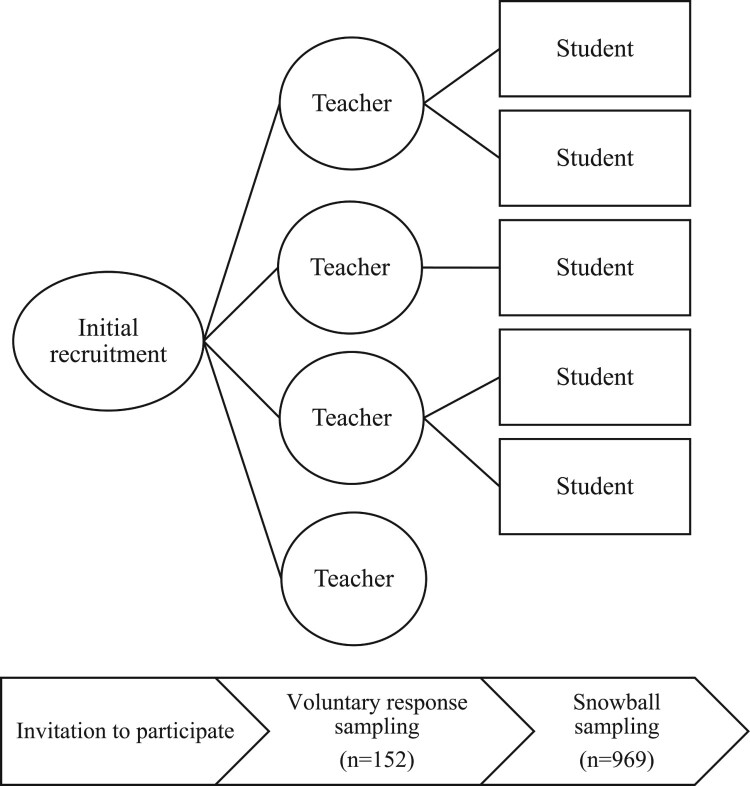
Sampling methods applied in the study.

**Figure 2.**
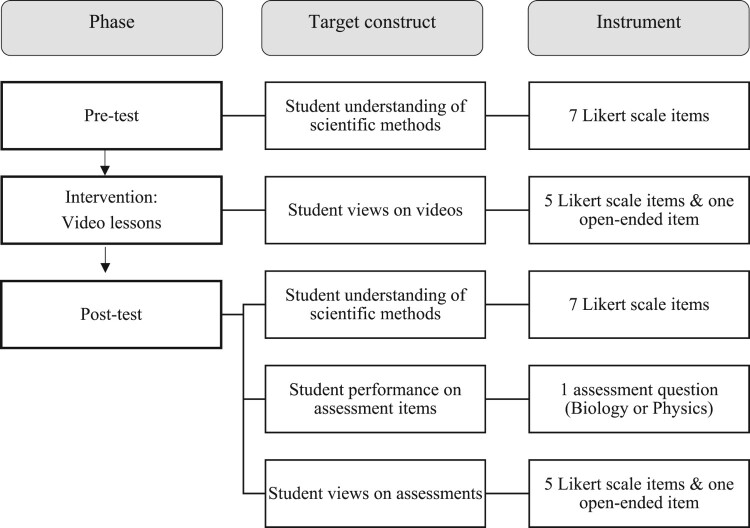
Overview of data collection phases, target constructs and instruments.

Data sources are responses from an online survey of secondary students’ understanding of the diversity of scientific methods implemented before and after watching online video lessons. The online videos and surveys were distributed to science teachers via email and social media across the United Kingdom, and the teachers subsequently voluntarily used the resources to teach their students online during the Covid-19 lockdown of Spring 2020 in the UK, during which schools were closed for face-to-face teaching. The sampling method was a combination of voluntary response and snowball sampling, which is preferred compared to convenience sampling, which may potentially bias outcomes (see Figure 1). Once teachers agreed to participate in the study, they received a set of resources through one link that included in sequence a survey, video lesson materials, summative assessment items and further surveys. The entire set was delivered by the teachers as part of their regular lessons and lasted for about an hour. From a research point of view, the set incorporated three distinct phases (see Figure 2). There was a pre-test that included a short survey of 7-Likert scale items that measured students’ understanding of scientific methods. Once the students completed the survey, they automatically moved onto the intervention, which included a set of videos. One video was an introduction to scientific practices and the second video was on a science topic in biology, physics or chemistry. The teachers instructed their students to choose one topic. After watching the videos, the students were asked to complete another 5-Likert scale items and one open-ended question. Subsequently, the survey used at the beginning as part of the pre-test was repeated, along with further questions on the science topic and the evaluation of the videos. What is referred to as the ‘intervention’ is the input about the scientific methods through the videos. In this paper, we focus on the analysis of the pre-test and post-test 7-Likert scale items. The online teaching videos (or video lessons) are the main feature of the intervention reported in relation to the research question. The summative assessments are used as indicators of student outcomes along with the data from the pre- and post-surveys on students’ understanding of scientific methods.

Overall, 969 students (Table 2) and 152 teachers (Table 3) participated in the study. The focus in this paper is on the students’ outcomes. The majority of the students were in Year 9 (13–14 years old). In England, the 2-year groups consisting of students aged 13–15 is referred to as Key Stage 4. This is the point at which students sit national examinations called GCSEs. The outcomes of their examinations pave the way for their applications to university. In that sense, they are high-stakes examinations. Given that the majority of the sample (total of about 68%) were Key Stage 4 students, it can be said that the outcomes of the study can have direct implications for the actual national context of high-stakes examinations. 50.3% were male, 46.3% female and 3.4% preferred not to declare their gender. According to their teachers, the majority was from South East of England and from Academies (Table 4). Academies are state-funded schools that sometimes have a private sponsor. Most secondary schools (high schools) in England are now Academies.

**Table 2.** Age groups of students in the study.

Age	Frequency	Per cent
Year 6 and under	1	0.1
Year 7	116	12
Year 8	176	18.2
Year 9	426	44
Year 10	229	23.6
Year 11	17	1.8
Year 12 and above	4	0.4
Total	969	100

**Table 3.** Geographical distribution of teachers.

Region	Frequency	Percentage
Northern Ireland	3	0.3
North East	64	6.6
North West	12	1.2
Yorkshire and the Humber	94	9.7
West Midlands	68	7
East Midlands	16	1.7
South West	84	8.7
South East	278	28.7
East of England	124	12.8
Greater London	161	16.6
Other	65	6.7
Total	969	100

**Table 4.** Types of schools represented in the sample.

Type of school	Frequency	Percentage
Academy	312	32.2
State	241	24.9
Faith	26	2.7
Private	194	20
Grammar	148	15.3
Other	48	5
Total	969	100

### Teaching intervention through videos

Brandon’s Matrix guided the development of four online lessons. A set of videos in physics, biology and chemistry topics were produced by those members of the team who are science teachers with research experience. The production of videos was done by the team using project camcorders and also edited by the team. In other words, the videos were not produced professionally because the time frame of the production coincided with the onset of the Covid-19 pandemic when social interaction was not allowed. During the planning of the lessons, the instructors used lesson plan templates and met regularly to discuss the pedagogical content (e.g. the learning objectives), as well as the practical issues that arose (e.g. needs for equipment). The instructors developed two kinds of videos. The first lesson session was an introduction to scientific methods using Brandon’s Matrix as guiding a framework. This was followed by the second type of lesson session, which was a presentation of practical investigation on a topic in either biology, chemistry or physics situated in an example matrix category such as hypothesis testing or parameter measurement. All videos made explicit reference to Brandon's categories and thus provided a meta-narrative to the investigation. This is in line with arguments for the explicit teaching of the nature of science (Lederman & Lederman, 2019), particularly given the effectiveness of the explicit teaching approach demonstrated in studies linking the nature of science to particular aspects of scientific reasoning (Khishfe, 2014). Such use of meta-language about scientific methods using Brandon’s Matrix reinforced the epistemic framing of the scientific methods represented in the videos.

In the video lessons, the instructors presented Brandon’s Matrix as a way to categorise and understand scientific methods. Students were thus shown two video-based lessons, as follows: Lesson 1 aimed to familiarise students with Brandon’s Matrix, while Lesson 2 aimed to present one of the three designed practical investigations. In other words, each student cohort only did one of the science subjects as the whole set would take a fairly long time to complete, particularly given the pre- and post-test surveys, including the assessments. The topics were an electromagnetic spectrum, osmosis and chromatography. Each lesson lasted an average of 10 min. Table 5 summarises the content of the video lessons. The ‘assessments’ were summative assessment items designed by a group of professional examiners affiliated with different examination boards in England. In a separate study, we reported about the summative assessments (El Masri et al., 2021). In the context of the present study, the summative assessments are used to illustrate the students’ performance given the teaching intervention through the videos. Both the examiners and the team members who produced the videos were trained about Brandon’s Matrix through a set of professional development workshops. In these workshops, the participants were introduced to Brandon’s Matrix, and they applied the framework to sets of practicals that are typical for the age group in the national science curriculum in England.

**Table 5.** The description of online teaching videos.

**Video lessons** (20–25 min)			
**Lesson 1** (all students)	**Lesson 2** (choose between)		
*Introduction to scientific methods*	*Electromagnetic spectrum*	*Osmosis*	*Chromatography*
• This video presents examples of how scientists choose from a variety of scientific methods to investigate a question or a claim (e.g. ‘The day and night are caused by a spinning earth.’)	• The practical investigation of the electromagnetic spectrum is presented using the example of refraction.	• The practical investigation of osmosis is presented to examine the effect of a range of concentrations of sugar solutions on the mass of plant tissue.	• The practical investigation of chromatography is presented as a technique to examine what coloured compounds are used to make purple ink.
• Foucault’s pendulum is discussed as an example of non-manipulative hypothesis testing	• The instructor discusses how the followed procedure fits into the *manipulative parameter measurement.*	• The instructor discusses how the investigation fits in the *manipulative parameter measurement* category.	• The instructor discusses how the investigation fits into the *non-manipulative parameter measurement* category.
• The video introduces Brandon’s Matrix as a way to categorise scientific methods	• Examples of other investigations involving the phenomenon of the electromagnetic spectrum are discussed.	• Examples of other investigations involving the phenomenon of osmosis are discussed.	• Examples of other investigations involving chromatography are discussed.
• The video provides examples for each category of matrix categories			

### Research instrument

In order to examine whether or not the designed intervention in the form of online videos would facilitate students’ understanding of scientific methods, an online questionnaire was developed targeting students’ understanding of scientific methods before and after the intervention. The online questionnaire consisted of seven five-point Likert scale questions, which were a combination of new and adapted items used in previous research to measure students’ nature of science (NOS) views. Three of these items targeted students’ general understanding of scientific methods, while four aimed to measure students’ understanding regarding the scientific methods as epistemologically framed in Brandon’s Matrix. Table 6 presents the used items and the targeted constructs.

**Table 6.** Survey questions targeting students’ understanding of scientific methods.

Item	Construct	1	2	3	4	5
2.1 There is a universal scientific method that scientists follow.	General	Strongly agree	Agree	Neutral	Disagree	Strongly Disagree
2.2 In science investigations follow step-by-step procedures.	General	Strongly agree	Agree	Neutral	Disagree	Strongly Disagree
2.3 An experiment is not always the best way to test a hypothesis.	Brandon’s Matrix-specific	Strongly Disagree	Disagree	Neutral	Agree	Strongly Agree
2.4 In order to be scientific, an investigation should include hypothesis testing	Brandon’s Matrix-specific	Strongly agree	Agree	Neutral	Disagree	Strongly Disagree
2.5 In order to be scientific, an investigation should include the manipulation of a variable.	Brandon’s Matrix-specific	Strongly agree	Agree	Neutral	Disagree	Strongly Disagree
2.6 Observations that do not include hypothesis testing or manipulation of a variable are not scientific investigations.	Brandon’s Matrix-specific	Strongly agree	Agree	Neutral	Disagree	Strongly Disagree
2. 7 Science does not always follow a universal method.	General	Strongly Disagree	Disagree	Neutral	Agree	Strongly Agree

The items that were used to measure students’ general understanding of scientific methods were adapted items from previous research. More explicitly, item 2.1 (‘There is a universal scientific method which scientists follow.’) was adapted from a scoring rubric previously used by Kartal et al. (2018). Similarly, item 2.2 (‘In science investigations follow step-by-step procedures.’) was previously used by Lederman and Lederman (2019) (VOSI, item 3c). These items were selected because, in line with Brandon’s Matrix, they targeted students’ views of ‘the scientific method.’ The items targeting students’ understanding of scientific methods as presented in the matrix were developed for the needs of the present study. More explicitly, four items were constructed to capture students’ understanding of the two basic components of Brandon’s Matrix (hypothesis testing and manipulation of a variable: items 2.4 & 2.5), as well as their views on the two ‘extreme cases’ of scientific investigations (manipulative hypothesis testing and non-manipulative parameter measurement: items 2.3 & 2.6).

In addition, five Likert scale items were constructed to examine students’ views on the online lessons. The questions aimed to investigate whether students viewed the online lessons as useful resources for learning about practical science. More explicitly, students were asked if they found the online lessons helpful for learning about scientific methods and whether they were able to facilitate understanding of the specific practical that was presented. Finally, the students were asked whether they enjoyed watching the videos and whether the demonstrations were similar to the ones that they performed in their classrooms. Thus, these questions served a second purpose; by eliciting students’ views on the online lessons, we would be able to ensure the ecological validity of the designed intervention.

## Results and findings

The results and findings are summarised with respect to the (a) students’ views about the teaching videos and the related summative assessments; (b) impact of the videos on students’ understanding of the diversity of scientific methods; and (c) students’ performance on a pair of summative assessment items focusing on manipulative parameter measurement in the context of biology and physics topics. The first set of results are important to note given the significance of students’ views and attitudes towards lesson resources and assessments in effective incorporation of strategies in teaching and learning (Osborne & Collins, 2001). The second set of results targets the effectiveness and the impact of the intervention and addresses the key research question in terms of the extent to which the epistemic framing of video lessons might have had an impact on students’ understanding of scientific methods. Finally, the students’ responses to the summative assessments provide an indication on how the students performed on some example examination-like questions. Considering the various permutations of assessments that can be analysed in the whole data set, it is beyond the scope of this paper to present a full account of how the students performed on all assessment items. Rather, here we illustrate how the students performed on some key elements of the Brandon Matrix categories, namely the manipulation of variables in the parameter measurement category.

**Table 7.** Students’ views on online teaching videos.

	Biology	Chemistry	Chemistry
I think that the videos were about practical science	77%	78%	78%
I enjoyed watching the videos	43%	39%	39%
The demonstrations that were included in the videos were similar to demonstrations or investigations that I normally do in class	68%	81%	73%
Session 1 helped me understand scientific methods better	75%	69%	81%
Session 2 helped me understand the topic better	67%	73%	68%

### Students’ views of the teaching videos and summative assessments

The results suggest that the majority of the students indicated that the resources helped them understand scientific methods (see Table 7). Among the science topics, chromatography was ranked the highest in terms of the videos helping the students to understand the topic better. Eighty-one per cent of the students involved in the physics activities indicated that the session on the introduction to Brandon’s Matrix helped them understand scientific methods better. One student remarked:

Before I watched the videos, I thought all scientific investigations require hypothesis of some sort, which these videos disproved in an easy to understand way. Thank you for opening my eyes.

However, the students were less keen on watching the videos across all the science topics. One 39% of the students enjoyed the physics and chemistry videos, whereas the proportion of students enjoying the biology videos was higher at 43%. When asked to provide feedback through open-ended questions, one student remarked:

I personally find it easier to understand information when a person is saying it to me as I find it easier to concentrate and listen but I think the videos did a good job of explaining the concepts.

The student’s comments seem to be more about the lack of a direct personal involvement of a teacher rather than the content of the videos. This finding is in line with previous research suggesting that learners find face-to-face laboratory sessions more enjoyable than online sessions (e.g. Brockman et al., 2020; Salter & Gardner, 2016), as they are hesitant to accept alternative leaning modalities (Brockman et al., 2020)*.* This could also be interpreted in the context of the study which was done at the onset of the Covid-19 pandemic when schools had quite suddenly switched to online platforms during the first lockdown in England. It could be that the students were saturated by online exposure to lessons. However, further data would be needed to understand what attitudes students would have to the videos had the circumstances been different. Similarly, although they were mostly positive about the videos helping them answer the assessments, they were less keen about doing the assessments (see Table 8).

They were quite challenging. However if we had learnt more about this topic in lessons, I personally would have found them easier.

**Table 8.** Students’ views on assessments.

	Biology	Chemistry	Chemistry
I enjoyed completing the questions	38%	27%	42%
I think that the questions were about practical science	82%	82%	82%
I found the questions challenging	35%	49%	29%
The videos helped me answer the questions	63%	57%	57%
The questions were similar to the questions that I normally complete in class for GCSE	56%	48%	62%

Not surprisingly, the students were not keen on the assessments (see Table 8). However, they did characterise all videos to be about practical science, which provided confirmation from students’ point of view that the assessment items were indeed about the construct of practical science. Nearly half of the students thought that the assessment items were similar to what they would normally do in their regular lessons. This finding suggests that the students did recognise that the items had a different way of framing the topics that they would normally cover.

### The impact of the teaching videos on students’ understanding of scientific methods

Even though the students were not very keen on the videos and the assessments, the findings illustrate that the intervention made a statistically significant impact on their understanding of scientific methods, including across all science topics. The particular aspects of scientific methods, such as the universality of scientific methods (or not), were also statistically significant for all topics (see Table 9). Furthermore, although improvement of conceptual understanding was not a goal of the project, there were some improvements in students’ understanding in the context of osmosis and electromagnetism. For instance, a higher number of correct answers were observed about the definition of osmosis (70% versus 63%), the concentration of sugar solutions (45% versus 35%) and specular reflection (70% versus 65%). The frequency of correct responses was similar in the context of chromatography as well as some other concepts such as absorption and reflection. It should be noted that prior to the intervention, some students had not done a practical in chromatography (19%), osmosis (40%) and electromagnetic spectrum (22%). This background information might explain why the gains in the context of osmosis were more striking, whereas previous exposure to practicals might have resulted in students losing interest in the videos and the assessments even though they were receptive to the epistemic framing of the practical given the statistically significant outcome in their understanding of scientific methods.

**Table 9.** Paired samples t-test for items measuring students’ understanding of scientific methods.

	Pre-test		Post-test			
	Mean	SD	Mean	SD	t	*p*
(1) There is a universal scientific method that scientists follow.	2.55	0.96	2.90	1.17	9.83	<.001*
(2) In science, all investigations follow step-by-step procedures.	2.07	0.92	2.50	1.05	13.20	<.001*
(3) An experiment is not always the best way to test a hypothesis.	2.98	0.95	.3.27	0.94	8.18	<.001*
(4) In order to be scientific, an investigation should include hypothesis testing.	2.43	0.67	3.13	1.17	16.19	<.001*
(5) In order to be scientific, an investigation should include the manipulation of a variable.	2.45	0.98	3.25	1.7	17.93	<.001*
(6) Observations that don’t include hypothesis testing or the manipulation of a variable are not scientific investigations.	3.05	1.02	3.51	1.11	11.62	<.001*
(7) Science does not always follow a universal method.	3.55	0.94	3.66	0.97	3.21	.001*

Note: *p < .01, SD = Standard Deviation.

### Students’ performance on summative assessments in physics and biology topics

Students completed summative assessment questions on all science topics. For the purposes of our discussion here, we focus on manipulative parameter measurement examples from biology and physics as they provide a consistent account of one Brandon’s Matrix category. In other words, the purpose of this analysis is to provide examples of how students performed on the assessment questions in different topics of the same Brandon’s Matrix category. The assessments were in line with the videos in given the key focus of the investigations in the videos was manipulative parameter measurement as well. Given the finding that the videos had a statistically significant impact on students’ understanding of scientific methods, we were also interested in finding out how the students performed on the assessment items themselves. Considering the various permutations of assessments that can be analysed, it is beyond the scope of this paper to present a full account of how the students performed on all assessment items. Rather, here we illustrate how the students performed on some key elements of the Brandon Matrix categories, namely the manipulation of variables in the parameter measurement category.

**Figure 3.**
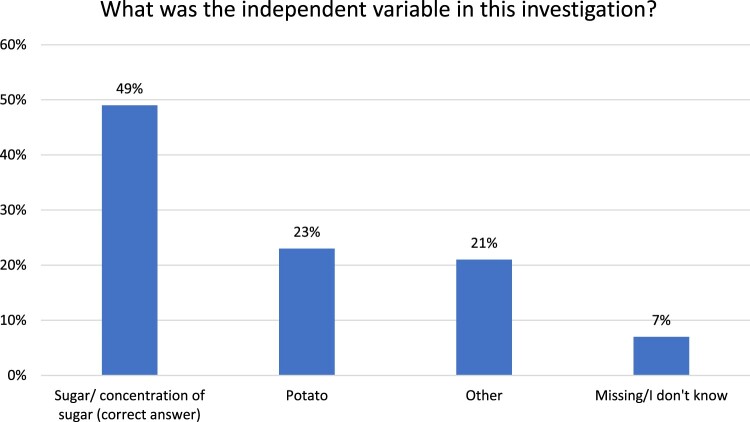
Student assessment data from the osmosis scenario.

In the case of the biology topic, osmosis, there were three questions as illustrated in the Appendix. The students were presented with a scenario of osmosis where potato chips are placed in different concentrations of sugar solutions. The steps of the investigation are provided, and the students are then asked to identify the variables involved. Two of the three questions had an epistemic dimension in terms of variables, while the third question focused on mathematics skills (see the Appendix). When the data from the sample of students (n = 289) were analysed, it was found that the majority of the students (49%) were able to identify the sugar solution as a key independent variable in the investigation (see Figure 3). Some of the responses were either irrelevant or were incomplete. For instance, sometimes, the students did not provide a full statement about ‘concentration of sugar solution’ but rather only referred to ‘sugar’ or ‘concentration.’ Such instances were not taken to be the correct response and were included in the ‘other’ category. It could be that some of these responses were also indicative of understanding but that the students did not feel that they needed to provide a full reference to the variable. In any case, the results indicate that the majority of the students were able to identify the variables involved in the investigation. Considering the osmosis scenario was an instance of manipulative parameter measurement, the focus on the variables was a central aspect of the assessment questions.

**Figure 4.**
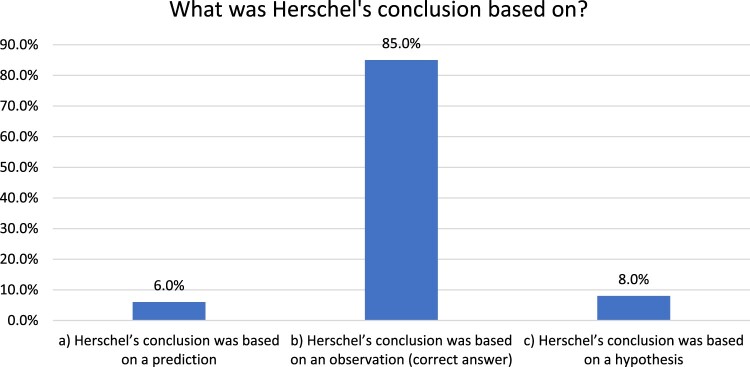
Student assessment data from the electromagnetism scenario.

In the case of the physics topic, electromagnetism, the students were provided with a historical case study about the eighteenth-century scientist William Herschel’s investigation on the heating effects of different colours of light. The students were shown a diagram and given data on the differences in thermometer readings in different parts of the spectrum. There were three questions (see the Appendix). One of the questions asked students to identify the type of methodological approach (e.g. prediction, observation, hypothesis) that Herschel’s conclusion was based on. When the data of the sample of students (n = 251) were analysed, the majority of the students (85%) correctly identified the methodological approach (see Figure 4). Overall, the assessment questions were not unlike what they would be in actual high-stakes examinations, hence providing utility for real adaptation in educational systems.

Overall, then, one assessment item from the biology and one from the physics questions were analysed for a total of 540 students. Both items had different elements in epistemic framing: one in terms of manipulation of variables and the other in terms of the methodological approach. Hence collectively, they provide an indication of the students’ performance on the epistemic aspects of scientific methods. Considering the various permutations of how the assessment items can be compared, as well as the large sample of students, it is not possible to provide a full account of the assessment data in this paper. However, the findings from Figures 3 and 4 provide some indication of the impact of the intervention on the students’ performance on the assessment questions in relation to how they applied their understanding in a written format, not unlike an actual high-stakes examination setting.

## Conclusions and discussion

In this paper, we argued that epistemic framing of science lessons is an important goal and illustrated an empirical study on how to align curriculum content and summative assessment with epistemic goals. We have taken on a well-justified and theoretised construct of scientific methods based on a philosopher’s account (Brandon, 1994), which has already been applied empirically in other contexts, for instance, in the analysis of examination questions (Cullinane et al., 2019) and used it as a guiding framework to generate coherence between teaching videos and summative assessment items. We have subsequently conducted an empirical investigation where we traced the impact of an online teaching and summative assessment intervention on students’ understanding of Brandon’s Matrix. Using a theoretical framework on the diversity of scientific methods ranging from hypothesis testing to non-manipulative parameter measurement, video lessons and summative assessments were designed and tested with secondary teachers and students in England. Our approach consisted of explicit integration of Brandon’s Matrix as a way to empirically frame the practical activities in teaching videos. Such an approach is consistent with the literature that has provided evidence for the need for explicit teaching of the nature of science (Khishfe, 2014; Lederman & Lederman, 2019).

The findings suggest that the students’ understanding of scientific methods significantly improved after watching the videos. Furthermore, the students’ performance on the summative assessment items indicated a high level of accuracy in responses. The majority of the national sample of 969 students (or a total of about 68%) were Key Stage 4 students, and the sample size of the teacher cohort was fairly large and diverse, it can be said that the outcomes of the study have direct implications for the actual national high-stakes examinations.

Although epistemic practices of science have been advocated as an important component of science education for decades (Kelly & Takao, 2002) there have been few examples of coherent teaching and learning resources coupled with examples of high-stakes assessments. Identifying frameworks such as Brandon’s Matrix that constitute simple heuristics for framing typical science activities from an epistemic perspective is a critical step in designing and testing the impact of innovative science teaching and summative assessment. Given assessment practices, particularly summative assessments influence how the science curriculum is experienced by students, it is imperative for educational systems to strive for coherence between curriculum and assessment. In the empirical study presented in the paper, we have demonstrated how a theoretically well formulated and justified framework can be utilised for the design and evaluation of teaching and assessment approaches. The assessment questions were not unlike what they would be in actual high-stakes examinations, hence providing utility for real adaptation in educational systems. The epistemic framing of such teaching and assessment approaches in the context of scientific methods is important given the problematic history of the teaching of the scientific method in science education. While many sources in the science education literature acknowledge that there is no single scientific method that operates in a stepwise fashion (e.g. Woodcock, 2014), further research is needed to understand how to equip students with the understanding of the plurality of methods in science. The study provides some concrete examples of how the diversity of scientific methods can be framed from an epistemic perspective in teaching and assessment and provides evidence for their effectiveness.

## Appendix

### Part 1: Osmosis

A student investigated the effect of placing potato chips in different concentrations of sugar solutions. This is the method he used:

Give **two** variables the student should control in this investigation.

What was the independent variable in this investigation?

The mass of one of the potato chips was 4.1g at the start and 4.5g after being in the sugar solution. Calculate the percentage increase in mass of this potato chip.

### Part 2: Electromagnetic spectrum & refraction

In the 18th century, a scientist called William Herschel investigated the heating effect of different colours of light.

Figure A1 shows the equipment he used.

Herschel used a prism to split white light into a spectrum of colours.

He recorded the temperature increase using thermometers placed in four positions, **A**, **B**, **C** and **D.**

His results are shown in Table A1.

**Table A1**

Suggest **one** reason Herschel would have been surprised by the result for thermometer D.

Herschel concluded that there must be an invisible form of light beyond red in the visible spectrum. Suggest why.

What was Herschel’s conclusion based on? Tick **one** box.
